# ﻿Morphological and molecular data reveal *Cerrenacaulinicystidiata* sp. nov. and *Polyporusminutissimus* sp. nov. in Polyporales from Asia

**DOI:** 10.3897/mycokeys.106.121840

**Published:** 2024-06-13

**Authors:** Zi-Wei Zheng, Qiu-Yue Zhang, Li-Rong Zhang, Hai-Sheng Yuan, Fang Wu

**Affiliations:** 1 State Key Laboratory of Efficient Production of Forest Resources, School of Ecology and Nature Conservation, Beijing Forestry University, Beijing 100083, China Beijing Forestry University Beijing China; 2 Center for Biodiversity and Nature Reserve, Chinese Academy of Environmental Planning, Beijing 100043, China Chinese Academy of Environmental Planning Beijing China; 3 Institute of Applied Ecology, Chinese Academy of Sciences, Shenyang 110016, Liaoning, China Institute of Applied Ecology, Chinese Academy of Sciences Shenyang China

**Keywords:** Cerrenaceae, phylogeny, Polyporaceae, taxonomy, wood-decaying fungi

## Abstract

Two new species of Polyporales, *Cerrenacaulinicystidiata* and *Polyporusminutissimus*, are illustrated and described on the basis of morphological studies and phylogenetic analyses from southern China and Vietnam. *C.caulinicystidiata* is characterized by annual, resupinate, sometimes effused-reflexed basidiocarps, greyish orange to brownish orange pore surface, irregular pores (3–8 per mm), a trimitic hyphal system, pyriform to ventricose cystidia, and subglobose basidiospores 3.2–4.5 × 2.8–3.5 µm in size. *P.minutissimus* is characterized by annual, solitary, fan-shaped with a depressed center or infundibuliform basidiocarps, obvious black stipe, cream to buff yellow pileal surface with glabrous, occasionally zonate and radially aligned stripes, angular pores (6–9 per mm), a dimitic hyphal system, and cylindrical basidiospores, 5–9.2 × 2.2–4 μm. Detailed descriptions and illustrations of the two new species are provided. The differences between the two new species and their morphologically similar and phylogenetically related species are discussed.

## ﻿Introduction

The order Polyporales presents a great diversity of basidiocarp types and hymenophore configurations (Binder et al. 2013). It is an important group of fungi, as Polyporales species can cause wood-decay and thus play an essential role in the carbon cycle. In addition, some species of Polyporales may have medicinal properties (Dai 1996, 1999, 2012; Dai et al. 2009). The order has long been the subject of research on taxonomic diversity, distribution patterns, and ecological functions (Hibbett et al. 2014). As of early 2024, more than 1,800 species are recognized in the order (Martinez et al. 2004; Martinez et al. 2009; Kirk et al. 2008; Grigoriev et al. 2013; Zhao et al. 2015; Justo et al. 2017). Due to its great diversity, the order is intensively studied worldwide (Justo et al. 2017).

*Cerrena* Gray is the type genus of Cerrenaceae within the Polyporales (Ryvarden and Gilbertson 1993; Justo et al. 2017), and it is typified by *C.unicolor* (Bull.) Murrill. It is widely distributed throughout the world. The genus is characterized by resupinate, effused-reflexed or pileate basidiocarps, irregular hymenophore, dimitic or trimitic hyphal systems, cylindric to ellipsoid basidiospores, and white rot (Ryvarden and Gilbertson 1993; Ryvarden et al. 2022). The genus *Cerrena* published in 1821 has priority above *Trametes* Fr., published in 1838, and the two genera were considered as a single taxon in several studies. Thus, a huge number of new combinations in *Cerrena*. (Cunningham and Cunningham1965; Gilbertson and Ryvarden 1987; Corner 1989; Ryvarden and Melo 2022). However, Ryvarden (1991) recommended to keep *Cerrena* as a separate genus based on their anatomical characters. According to Index Fungorum (http://www.indexfungorum.org) and Yuan (2014), *Cerrena* currently comprises around 10 species.

*Polyporus* P. Micheli ex Adans., the type genus of the Polyporaceae, is a well-known polypore genus (Gilbertson and Ryvarden 1987). Given that Micheli (1729) did not originally select a type species for *Polyporus*, there is no consensus on the selection of the type. Since Donk (1933) selected *P.tuberaster* (Jacq. ex Pers.) Fr. as the type species, this lectotype was accepted by most subsequent mycologists (Cunningham and Cunningham 1965; Singer 1986; Niemelä and Kotiranta 1991; Ryvarden 1991; Sotome et al. 2008; Ji et al. 2022). Morphologically, *Polyporus* is a heterogeneous genus including many species belonging to six morphological groups described by Núñez and Ryvarden (1995), viz, the *Polyporus* group, the *Favolus* group (= *Favolus* Fr.), the *Melanopus* group (= *Melanopus* Pat.), the *Polyporellus* group (= *Polyporellus* Karst.), the *Admirabilis* group, and the *Dendropolyporus* group (= *Dendropolyporus* (Pouz.) Jülich). Phylogenetically, *Polyporus* s. str. is known as a polyphyletic genus (Krüger et al. 2006; Sotome et al. 2008, 2011; Ji et al. 2022). Phylogenetic analyses of *Polyporus* did not conform to the six morphological groups, for which further in-depth study of the group is needed (Sotome et al. 2008; Zhou et al. 2016).

During investigations on wood-decaying polypores from South China and Vietnam, specimens that morphologically fit the definitions of *Cerrena* and *Polyporus* were collected. Phylogenetically, these samples formed two distinct lineages within *Cerrena* and *Polyporus*, respectively, and they are different from their morphologically similar and phylogenetically related species. Therefore, we describe and illustrate two new species, *Cerrenacaulinicystidiata* sp. nov. and *Polyporusminutissimus* sp. nov. within the Polyporales on the basis of morphological studies and phylogenetic analyses.

## ﻿Materials and methods

### ﻿Morphological studies

The studied specimens are deposited in the Fungarium of Beijing Forestry University (BJFC) and the Institute of Applied Ecology of the Chinese Academy of Sciences (IFP). Macro-morphological descriptions were based on field notes and voucher herbarium specimens. Microscopic measurements and drawings were made from slides prepared from voucher tissues and stained with Cotton Blue and Melzer’s reagent. The following abbreviations were used: KOH = 5% potassium hydroxide; CB = Cotton Blue; CB+ = cyanophilous in Cotton Blue; CB– = acyanophilous in Cotton Blue; IKI = Melzer’s; IKI– = neither amyloid nor dextrinoid in Melzer’s reagent; L = mean basidiospore length (arithmetic average of basidiospores); W = mean basidiospore width (arithmetic average of basidiospores); Q = variation in the L/W ratios between specimens studied; n (a/b) = number of basidiospores (a) measured from the given number of specimens (b). When we present basidiospore size variation, 5% of measurements were excluded from each end of the range. These excluded values are given in parentheses. Special color terms follow Anonymous (1969) and Petersen (1996).

### ﻿DNA extraction and sequencing

A CTAB rapid plant genome extraction kit (Aidlab Biotechnologies, Co., Ltd., Beijing, China) was used to obtain DNA products from voucher specimens following the manufacturer’s instructions with some modifications (Wu et al. 2020, 2022). The following primer pairs were used to amplify the DNA: ITS5 and ITS4 for the internal transcribed spacer (ITS) region (White et al. 1990) and LR0R and LR7 for the nuclear large subunit (nLSU) rDNA gene (Vilgalys and Hester 1990).

The procedures for DNA extraction and polymerase chain reaction (PCR) used in this study were the same as described by Wu et al. (2022). The PCR products were purified and sequenced by Beijing Genomics Institute (BGI), China. All newly generated sequences in this study were deposited in GenBank (Sayers et al.2024; http://www.ncbi.nlm.nih.gov/genbank/) and listed in Table 1.

**Table 1.** Taxa information and GenBank accession numbers of the sequences used in this study.

Species	Specimen No.	Country	ITS	LSU
*Cerrenaalbocinnamomea*	Miettinen 10511	China	OR262168	OR262168
*Cerrenaalbocinnamomea*	NIBRFG0000102423	South Korea	FJ821532	–
*Cerrenaalbocinnamomea*	Dai 12892	China	KC485522	KC485539
*Cerrenaalbocinnamomea*	KUC20121102-06	South Korea	KJ668561	–
***Cerrenacaulinicystidiata***	**Yuan 12664**	**Vietnam**	**MT269762**	**MT259328**
***Cerrenacaulinicystidiata***	**Yuan 12666**	**Vietnam**	**MT269763**	**MT259329**
*Cerrenacaulinicystidiata* (*Cerrena* sp. 1)	BJ2-11	China	KX527879	–
*Cerrenacaulinicystidiata* (*Cerrena* sp. 1)	G1669	China	MK247953	–
*Cerrenacaulinicystidiata* (*Cerrena* sp. 1)	Otu0185	China	MT908560	–
Cerrenacaulinicystidiata*	**Wu 661**	**China**	**PP035831**	**PP035828**
*Cerrenacystidiata*	548/17	Brazil	MZ649034	MZ649034
*Cerrenagilbertsonii*	JV 1609/29	Guadeloupe	OR262202	–
*Cerrenagilbertsonii*	Vandevender 94-144	Mexico	OR262171	OR262171
*Cerrenamultipileata*	JV 1407/63	Costa Rica	OR262201	OR262201
*Cerrenamultipileata*	Ryvarden 43881	Costa Rica	OR262155	OR262155
*Cerrenamultipileata*	Kout A36	Guatemala	OR262203	–
*Cerrena* sp. 2	F12	China	OP022000	–
*Cerrena* sp. 2	7-SU-3-B-77(M)-B	Indonesia	KJ654531	–
*Cerrena* sp. 2	NTOU5117	Taiwan	MN592928	–
*Cerrenaunicolor*	B2	Antarctica	HM589361	–
*Cerrenaunicolor*	D.T6.5_2	Argentina	MH019790	–
*Cerrenaunicolor*	CBS 154.29	Canada	MH855029	–
*Cerrenaunicolor*	He6082	China	OM100740	OM083972
*Cerrenaunicolor*	GSM-10	China	JQ798288	–
*Cerrenaunicolor*	Han 849	China	MW467890	–
*Cerrenaunicolor*	CU2	Czech	FJ821536	–
*Cerrenaunicolor*	H:Otto Miettinen 9443	Finland	FN907915	FN907915
*Cerrenaunicolor*	MUT<ITA_:5063	Italy	MK581063	–
*Cerrenaunicolor*	FCG-1937	Japan	LC415531	–
*Cerrenaunicolor*	Pertti Uotila 47558 (H)	Kyrgyzstan	OR262167	–
*Cerrenaunicolor*	Feketic	Serbia	MW485440	–
*Cerrenaunicolor*	KA17-0024	South Korea	MN294859	–
*Cerrenaunicolor*	3115	Sweden	JN710525	JN710525
*Cerrenaunicolor*	CUZFVG179	Turkey	MK120293	–
*Cerrenaunicolor*	K(M):249944	UK	MZ159683	–
*Cerrenaunicolor*	FD-299	USA	KP135304	KP135209
*Cerrenaunicolor*	TASM: YG/PS79	Uzbekistan	MT526291	–
*Cerrenazonata*	Gates 2008-4-17 (H)	Australia	OR262160	OR262160
*Cerrenazonata*	Otto Miettinen 9773 (H)	China	OR262157	OR262157
*Cerrenazonata*	Otto Miettinen 9889 (H)	China	OR262158	OR262158
*Cerrenazonata*	Otto Miettinen 13798 (H)	Indonesia	OR262166	OR262166
*Cerrenazonata*	WS36_1_2_B_As	Japan	LC631683	–
*Cerrenazonata*	PDD:95790	New Zealand	HQ533016	–
*Cerrenazonata*	KA17-0224	South Korea	MN294861	–
*Cerrenazonata*	LE-BIN 4492	Vietnam	OP985107	–
*Datroniellascutellata*	RLG9584T	USA	JN165004	JN164792
*Datroniellatropica*	Dai 13147	China	KC415181	KC415189
*Echinochaetebrachypora*	TFM:F 24996	Japan	AB462321	AB462309
*Echinochaeterussiceps*	TFM:F 15716	Japan	AB462310	AB368065
*Echinochaeterussiceps*	TFM:F 24250	Japan	AB462313	AB462301
*Favolusacervatus*	Cui 11053	China	KU189774	KU189805
*Favolusacervatus*	Dai 10749b	China	KX548953	KX548979
*Favolusgracilisporus*	Cui 4292	China	KX548970	KX548992
*Favolusgracilisporus*	Li 1938	China	KX548971	KX548993
*Hexagoniaglabra*	Dai 10691	China	JX569733	JX569750
*Hexagoniatenuis*	Cui 8468	China	JX559277	JX559302
*Irpexlatemarginatus*	Dai 8289	China	KY131835	–
*Lentinuslongiporus*	DAOM:229479	Canada	AB478880	LC052217
*Lentinuslongiporus*	WD2579	Japan	AB478879	LC052218
*Lentinussubstrictus*	Wei 1582	China	KU189767	KU189798
*Lentinussubstrictus*	Wei 1600	China	KC572022	KC572059
*Microporusaffinis*	Cui 7714	China	JX569739	JX569746
*Microporusflabelliformis*	Dai 11574	China	JX569740	JX569747
*Mycoboniaflava*	CulTENN10256	Costa Rica	AY513570	AJ487934
*Mycoboniaflava*	TENN59088	Argentina	AY513571	AJ487933
Neodatroniagaoligongensis*	Cui 8055	China	JX559269	JX559286
Neodatroniasinensis*	Dai 11921	China	JX559272	JX559283
*Neofavoluscremeoalbidus*	Cui 12412	China	KX899982	KX900109
Neofavoluscremeoalbidus*	TUMH:50009	Japan	AB735980	AB735957
*Neofavolusmikawai*	Cui 11152	China	KU189773	KU189804
*Neofavolusmikawai*	Dai 12361	China	KX548975	KX548997
*Physisporinuslineatus*	JV_1008_18	Costa Rica	OM669902	–
*Physisporinuslineatus*	JV_1407_37	Costa Rica	OM669903	–
*Physisporinusvinctus*	JV0610_A31B-1	Mexico	JQ409460	–
*Physisporinusvinctus*	JV0610_A31B-2	Mexico	JQ409461	–
*Picipesailaoshanensis*	Cui 12585	China	KX900068	KX900183
Picipesailaoshanensis*	Cui 12578	China	KX900067	KX900182
*Picipesamericanus*	JV 0809-104	USA	KC572003	KC572042
Picipesamericanus*	JV 0509-149	USA	KC572002	KC572041
Picipesannularius*	Cui 10123	China	KX900060	KX900176
*Picipesatratus*	Dai 13375	China	KX900042	KX900158
Picipesatratus*	Cui 11289	China	KX900043	KX900159
*Picipesauriculatus*	Yuan 4221	China	KX900064	KX900180
Picipesauriculatus*	Cui 13616	China	KX900063	KX900179
*Picipesbadius*	Cui 10853	China	KU189780	KU189811
*Picipesbadius*	Cui 11136	China	KU189781	KU189812
*Picipesbaishanzuensis*	Cui 11395	China	KU189763	KU189794
Picipesbaishanzuensis*	Dai 13418	China	KU189762	KU189793
*Picipesbrevistipitatus*	Cui 11345	China	KX900074	KX900188
Picipesbrevistipitatus*	Cui 13652	China	KX900075	KX900189
*Picipescf.dictyopus*	Cui 11109	China	KX900025	KX900145
*Picipescf.dictyopus*	Cui 11092	China	KX900026	KX900146
*Picipesconifericola*	Cui 9950	China	KU189783	KU189814
Picipesconifericola*	Dai 11114	China	JX473244	KC572061
*Picipesdictyopus*	TENN 59385	Belize	AF516561	AJ487945
*Picipesfraxinicola*	Dai 2494	China	KC572023	KC572062
*Picipesfraxinicola*	Wei 6025	China	KC572024	KC572063
*Picipesmelanopus*	H 6003449	Finland	JQ964422	KC572064
*Picipesmelanopus*	MJ 372-93	Czech	KC572026	KC572065
Picipesnigromarginatus*	Cui 8113	China	KX900062	KX900178
*Picipespumilus*	Cui 5464	China	KX851628	KX851682
*Picipespumilus*	Dai 6705	China	KX851630	KX851684
*Picipesrhizophilus*	Dai 11599	China	KC572028	KC572067
*Picipesrhizophilus*	Dai 16082	China	KX851634	KX851687
*Picipessubdictyopus*	Cui 11220	China	KX900057	KX900173
*Picipessubdictyopus*	Cui 12539	China	KX900058	KX900174
*Picipessubmelanopus*	Dai 13294	China	KU189770	KU189801
*Picipessubmelanopus*	Dai 13296	China	KU189771	KU189802
*Picipessubtropicus*	Li 1928	China	KU189758	KU189790
Picipessubtropicus*	Cui 2662	China	KU189759	KU189791
*Picipessubtubaeformis*	Cui 10793	China	KU189753	KU189785
Picipessubtubaeformis*	Dai 11870	China	KU189752	KU189784
*Picipestaibaiensis*	Dai 5741	China	JX489169	KC572071
Picipestaibaiensis*	Dai 5746	China	KX196783	KX196784
*Picipestibeticus*	Cui 12225	China	KU189756	KU189788
Picipestibeticus*	Cui 12215	China	KU189755	KU189787
*Picipestubaeformis*	Niemela 6855	Finland	KC572036	KC572073
*Picipestubaeformis*	JV 0309-1	USA	KC572034	KC572072
*Picipesulleungus*	Cui 12410	China	KX900022	KX900142
*Picipesvirgatus*	CulTENN11219	Argentina	AF516581	AJ488122
*Picipesvirgatus*	CulTENN11406	Argentina	AF516582	AJ488122
Picipeswuyishanensis*	Dai 7409	China	KX900061	KX900177
*Podofomesmollis*	RLG6304sp	USA	JN165002	JN164791
*Podofomesstereoides*	Holonen	Finland	KC415179	KC415196
Polyporusauratus*	Dai 13665	China	KX900056	KX900172
*Polyporusaustrosinensis*	Cui 11140	China	KX900046	KX900162
Polyporusaustrosinensis*	Cui 11126	China	KX900045	KX900161
*Polyporuscuticulatus*	Cui 8637	China	KX851614	KX851668
*Polyporuscuticulatus*	Dai 13141	China	KX851613	KX851667
*Polyporusguianensis*	TENN 58404	Venezuela	AF516566	AJ487948
*Polyporusguianensis*	TENN 59093	Argentina	AF516564	AJ487947
Polyporushapalopus*	Yuan 5809	China	KC297219	KC297220
*Polyporushemicapnodes*	Cui 11259	China	KX851625	KX851679
*Polyporushemicapnodes*	Dai 13403	China	KX851627	KX851681
*Polyporuslamelliporus*	Dai 12327	China	KX851622	KX851676
Polyporuslamelliporus*	Dai 15106	China	KX851623	KX851677
*Polyporusleprieurii*	TENN 58579	Costa Rica	AF516567	AJ487949
Polyporusmangshanensis*	Dai 15151	China	KX851796	KX851797
***Polyporusminutissimus***	**Wu 970**	**China**	**PP035829**	**PP035826**
Polyporusminutissimus*	**Wu 971**	**China**	**PP035830**	**PP035827**
*Polyporusparvovarius*	Yuan 6639	China	KX900049	KX900165
*Polyporusparvovarius*	Dai 13948	China	KX900050	KX900166
*Polyporusradicatus*	DAOM198916	Canada	AF516584	AJ487955
*Polyporusradicatus*	TENN 58831	USA	AF516585	AJ487956
*Polyporus* sp.*1*	Cui 11071	China	KX851642	KX851695
*Polyporus* sp.*1*	Cui 11045	China	KX851643	KX851696
*Polyporus* sp.*2*	Dai 13585A	China	KX900055	KX900171
*Polyporussquamosus*	Cui 10394	China	KX851635	KX851688
*Polyporussquamosus*	Cui 10595	China	KU189778	KU189809
*Polyporussubvarius*	WD2368	Japan	AB587643	AB587638
Polyporussubvarius*	Yu 2	China	AB587632	AB587621
*Polyporustuberaster*	Dai 11271	China	KU189769	KU189800
*Polyporustuberaster*	Dai 12462	China	KU507580	KU507582
*Polyporusumbellatus*	Pen 13513	China	KU189772	KU189803
*Polyporusvarius*	Cui 12249	China	KU507581	KU507583
*Polyporusvarius*	Dai 13874	China	KU189777	KU189808
*Pseudofavoluscucullatus*	Dai 13584A	China	KX900071	KX900185
*Pseudofavoluscucullatus*	WD2157	Japan	AB587637	AB368114
*Trametesconchifer*	FP106793sp	USA	JN164924	JN164797
*Trameteselegans*	FP105679sp	USA	JN164944	JN164799
*Trametespolyzona*	Cui 11040	China	KR605824	KR605767

Notes: New sequences are in bold; “–” represents missing data; * represents type specimens

### ﻿Phylogenetic analysis

Phylogenetic trees of *Cerrena* and *Polyporus* were constructed using the two concatenated ITS1-5.8S-ITS2-nLSU sequences dataset, respectively, and phylogenetic analyses were performed with Maximum Likelihood (ML) and Bayesian Inference (BI) methods. New sequences generated in this study and reference sequences retrieved from GenBank (Table 1) were partitioned to ITS1, 5.8S, ITS2, nLSU and then aligned separately using MAFFT v.74 (Katoh et al. 2019; http://mafft.cbrc.jp/alignment/server/) with the G-INS-I iterative refinement algorithm and optimised manually in BioEdit v.7.0.5.3 (Hall 1999). The separate alignments were then concatenated using PhyloSuite v.1.2.2 (Zhang et al. 2020). Unreliably aligned sections were removed before the analyses, and efforts were made to manually inspect and improve the alignment. The data matrix was edited in Mesquite v3.70. *Irpexlatemarginatus* (Durieu & Mont.) C.C. Chen & Sheng H. Wu was used as an outgroup in the phylogenetic analysis of *Cerrena* (Parmasto and Hallenberg 2000). *Trametesconchifer* (Schwein.) Pilát, *T.elegans* (Spreng.) Fr. and *T.polyzona* (Pers.) Justo were selected as outgroups in the phylogenetic analysis of *Polyporus* (Ji et al. 2022). The final alignments and the retrieved topologies were deposited in TreeBASE (http://www.treebase.org) under accessions 31102, 31103.

RAxML 7.2.8 was used to infer ML trees for both datasets with the GTR+I+G model of site substitution, including estimation of Gamma-distributed rate heterogeneity and a proportion of invariant sites (Stamatakis 2006). The branch support was evaluated with a bootstrapping method of 1,000 replicates (Hillis and Bull 1993).

For BI, the best-fit partitioning scheme and substitution model were determined by using ModelFinder (Kalyaanamoorthy et al. 2017) via the “greedy” algorithm, branch lengths estimated as “linked” and AICc. The BI was conducted with MrBayes 3.2.6 in two independent runs, each of which had four chains for 20 million generations and started from random trees (Ronquist et al. 2012). Trees were sampled every 1,000 generations. The first 25% of the sampled trees were discarded as burn-in and the remaining ones were used to reconstruct a majority rule consensus and calculate Bayesian Posterior Probabilities (BPP) of the clades.

Phylogenetic trees were visualized using FigTree version 1.4.4 (Rambaut 2018). Branches that received bootstrap support (BS) for ML and BPPs greater than or equal to 75% (ML) and 0.95 (BPP) were considered significantly supported, respectively.

## ﻿Results

### ﻿Phylogenetic analyses

**Figure 1.**
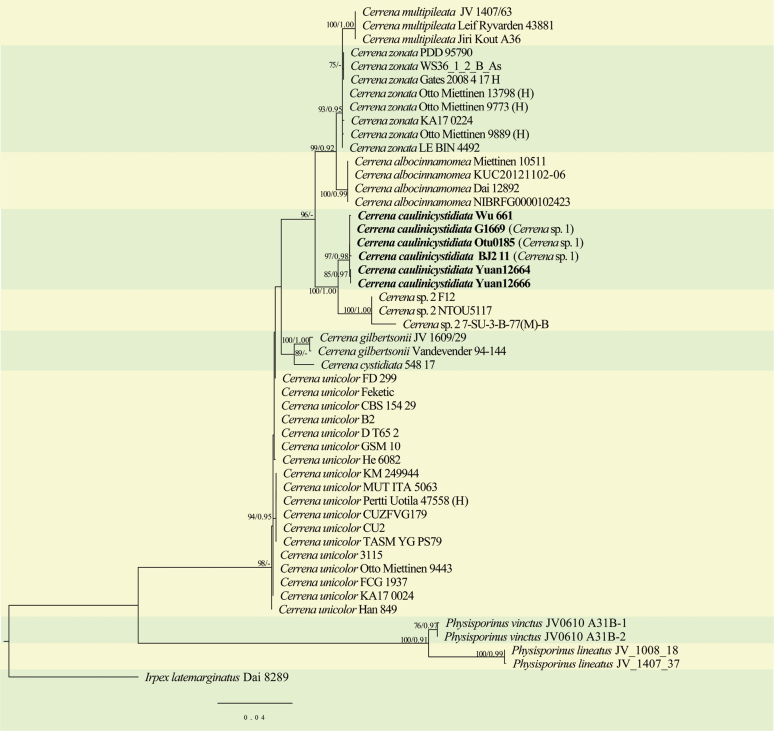
Maximum Likelihood (ML) phylogenetic tree illustrating the phylogeny of *Cerrena* and related genera in five families based on the combined ITS1-5.8S-ITS2-nLSU dataset. Branches are labeled with maximum likelihood bootstrap values (ML) higher than 75% and Bayesian posterior probabilities above 0.90. The new species is given in bold.

In the phylogenetic analysis of *Cerrena* (Fig. 1), the combined ITS1-5.8S-ITS2-nLSU dataset included sequences from 50 fungal collections representing 11 taxa, and one sample of *Irpexlatemarginatus* was used as an outgroup. ModelFinder proposed models were HKY+F+G4 for ITS1, GTR+F+I+G4 for 5.8s, HKY+F+G4 for ITS2 and GTR+F+I for nLSU, for Bayesian analysis. The BI analysis resulted in an average standard deviation of split frequencies = 0.008865. As both ML and BI trees resulted in similar topologies, only the topology of the ML analysis is presented together with the statistical values of the ML (≥75%) and BPP (≥0.90) algorithms (Fig. 1). The phylogeny inferred from ITS1-5.8S-ITS2-nLSU sequences (Fig. 1) showed that our three newly sequenced samples together with three samples defined as *Cerrena* sp. 1 by Miettinen et al. (2023) formed an independent lineage with strong support (97/0.98, Fig. 1). The lineage is defined as the new species *Cerrenacaulinicystidiata*.

**Figure 2.**
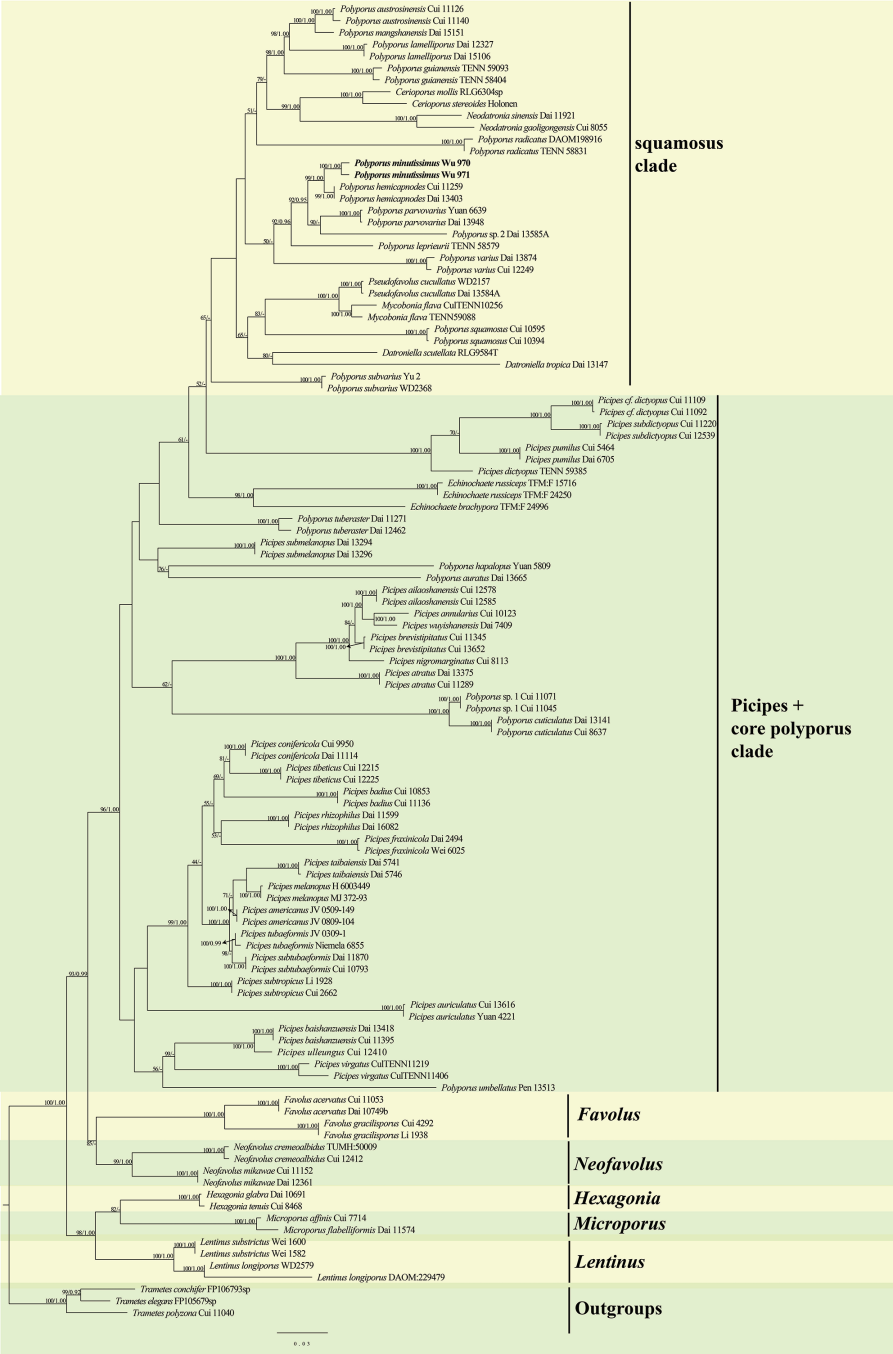
Maximum Likelihood (ML) phylogenetic tree illustrating the phylogeny of *Polyporus* and related genera based on the combined ITS1-5.8S-ITS2-nLSU dataset. Branches are labeled with maximum likelihood bootstrap values (ML) higher than 75% and Bayesian posterior probabilities (BPPs) more than 0.90. The new species is given in bold.

In the phylogenetic analysis of *Polyporus* (Fig. 2), the combined ITS1-5.8S-ITS2-nLSU dataset included sequences from 113 fungal collections representing 71 species, and three samples of *Trametes* were used as outgroups. ModelFinder suggested models were GTR+F+I+G4 for ITS1, K2P+I+G4 for 5.8s, K2P+I+G4 for ITS2 and GTR+F+I+G4 for nLSU, for Bayesian analysis. The BI analysis resulted in an average standard deviation of split frequencies = 0.009675. The ML and BI trees were similar in topology, and only the topology of the ML analysis is presented along with the statistical values of the ML (≥75%) and BPP (≥0.90) algorithms (Fig. 2). The phylogeny inferred from the combined ITS1-5.8S-ITS2-nLSU sequences (Fig. 2) revealed that a new lineage with high support (100/1.00,) nests in the squamosus clade in Polyporus, namely *Polyporusminutissimus*. The new species is closely related to *P.hemicapnodes* Berk. & Broome and *P.parvovarius* H. Lee, N.K. Kim & Y.W. Lim.

### ﻿Taxonomy

#### ﻿ Cerrena caulinicystidiata

Taxon classificationFungiPolyporalesCerrenaceae

T. Cao, F. Wu & H.S. Yuan sp. nov.

161AF221-198F-5B22-A0A7-DA9653A2DC30

853719

Figs 3 , 4

##### Holotype.

China • Zhejiang Province, Hangzhou, Xiaoshan District, Yangjingwu Forest Park; 30°4'1"N, 120°19'35"E; 134 m a.s.l.; 27 Mar. 2023; on fallen angiosperm branch; F. Wu leg., Wu 661 (BJFC040654).

##### Etymology.

*Caulinicystidiata* (Lat.): Refers to the cystidia with a tapering base.

##### Description.

***Basidiocarps*.** Annual, resupinate, sometimes effused-reflexed, continuous, easily separable, without special odor or taste when fresh, corky when dry, up to 10 cm long, 3 cm wide and 0.5 mm thick. Pore surface greyish orange to brownish orange; pores irregular, 3–8 per mm, partly split up to 2 mm long; dissepiments thin. Sterile margin finely fimbriated. Subiculum very thin, yellowish white, ca. 0.5 mm thick, a very thin brownish red crust present in the bottom next to wood. Tubes concolorous with pore surface, corky, 0.5–1 mm long.

***Hyphal structure*.** Hyphal system trimitic, generative hyphae with clamp connections; skeletal and binding hyphae CB+, IKI–; tissues unchanged in KOH.

***Subiculum*.** Generative hyphae thin- to slightly thick-walled, hyaline, clamped, frequently branched, 2–5 µm in diam; skeletal hyphae dominant, thick-walled to subsolid, unbranched, interwoven, 2.5–6 µm in diam; binding hyphae hyaline, thick-walled to subsolid, tortuose, moderately branched, 1.5–2.5 μm diam. The thin crust made up of subsolid, brownish and strongly agglutinated hyphae.

**Figure 3.**
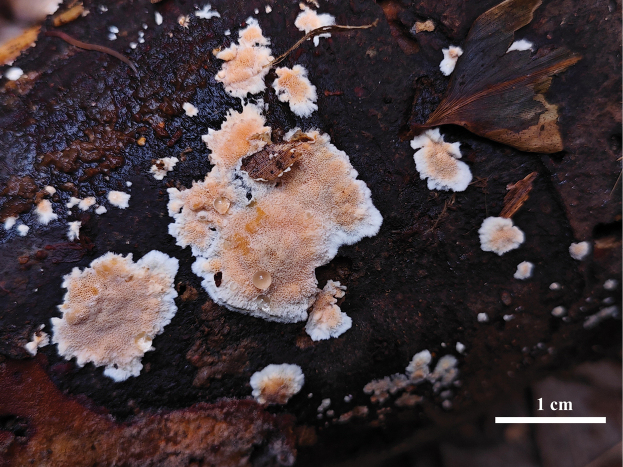
Basidiocarps of *Cerrenacaulinicystidiata* (Holotype, Wu 661).

***Tubes*.** Generative hyphae infrequent, hyaline, thin- to slightly thick-walled, clamped, rarely branched, 2–3 µm diam; skeletal hyphae dominant, hyaline, thick-walled to subsolid, rarely branched, sometimes with septate, interwoven, 2–4 µm in diam; binding hyphae rare. Cystidia clavate to pyriform to ventricose, mostly thin-walled, occasionally thick-walled, smooth, 13–20 × 6–12 µm; encrusted cystidia numerous, clavate, originated from and tightly embedded in trama, 10–25 × 7–15 µm (with encrustation). Basidia short clavate, with four sterigmata and a basal clamp, 8–11 × 4–5 µm, basidioles in shape similar to basidia, but slightly smaller.

**Figure 4.**
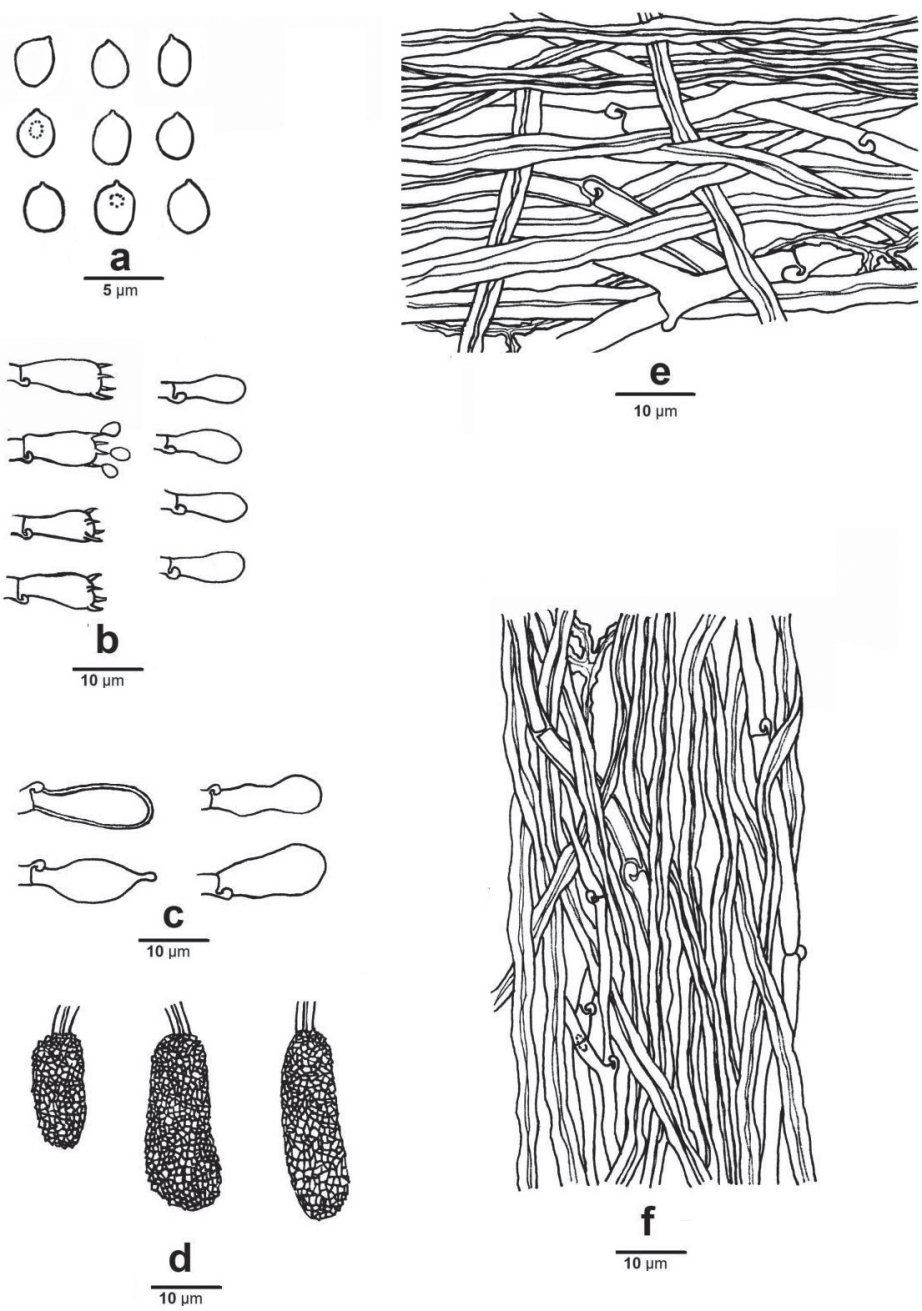
Microscopic structures of *Cerrenacaulinicystidiata* (Wu 661) **a** basidiospores **b** basidia and basidioles **c** cystidia **d** encrusted cystidia **e** hyphae from subiculum **f** hyphae from trama.

***Basidiospores*.** Basidiospores broadly-ellipsoid to ovoid, hyaline, thin-walled, smooth, CB–, IKI–, (3–)3.2–4.5(–4.8) × (2.5–)2.8–3.5(–3.9) µm, L = 3.94 µm, W = 2.84 µm, Q = 1.38–1.44 (n = 60/2).

##### Additional specimens examined

**(paratypes).** Vietnam • Lam dong Province (Lat.), Lac Duong District, Bidoup Nui Ba National Park; 12°11'8"N, 108°40'41"E; 1495 m a.s.l.; 15 Oct. 2017; on fallen angiosperm branch; H.S. Yuan leg., Yuan 12666 (IFP 019379), Yuan 12664 (IFP 019378).

#### ﻿ Polyporus minutissimus

Taxon classificationFungiPolyporalesPolyporaceae

Q.Y. Zhang, Z.W. Zheng & F. Wu sp. nov.

3C5B563B-D60C-5F05-A739-E3B1EC5F46A6

853720

Figs 5 , 6

##### Holotype.

China • Zhejiang Province, Hangzhou, Yuhang District, Luniao Town; 30°25'50"N, 119°42'38"E; 158.47 m a.s.l.; 9 Jun. 2023; on ground of Bamboo forest; F. Wu leg., Wu 971 (BJFC040963, holotype).

##### Etymology.

*Minutissimus* (Lat.): Referring to the species having tiny basidiocarps.

##### Description.

***Basidiocarps*.** Annual, centrally stipitate, solitary, fleshy to soft leathery when fresh, becoming fragile when dry. Pilei flat with a depressed center or infundibuliform, up to 1.5 cm in diam and 0.5–1 mm thick. Pileal surface cream to buff yellow when dry, glabrous, occasionally zonate and with radially aligned stripes; margin sharp, incurved upon drying. Pore surface cream when dry; pores angular, 6–9 per mm; dissepiments thin, entire. Context buff cream to pale neutral when dry, fragile upon drying, up to 0.5 mm thick. Tubes white to cream when dry, decurrent, up to 0.5 mm thick. Stipe dark violet, glabrous, 0.3–0.5 cm long and 1–2 mm in diam.

**Figure 5.**
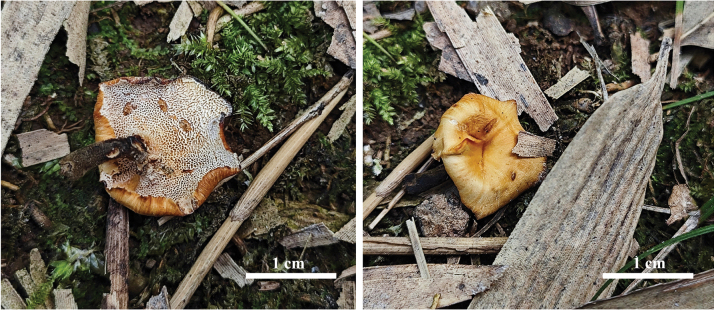
Basidiocarps of *Polyporusminutissimus* (Holotype, Wu 971).

***Hyphal structure*.** Hyphal system dimitic; generative hyphae bearing clamp connections, thin-walled, hyaline; skeleton-binding hyphae thick-walled with a wide lumen, with arboriform branches, IKI–, CB+; tissue unchanged in KOH.

***Context*.** Generative hyphae frequent, colorless, thin-walled, 2.5–4 μm in diam; skeleto-binding hyphae dominant, colorless, thick-walled with a wide lumen, moderately branched, strongly interwoven, 2–4.5 μm diam.

**Figure 6.**
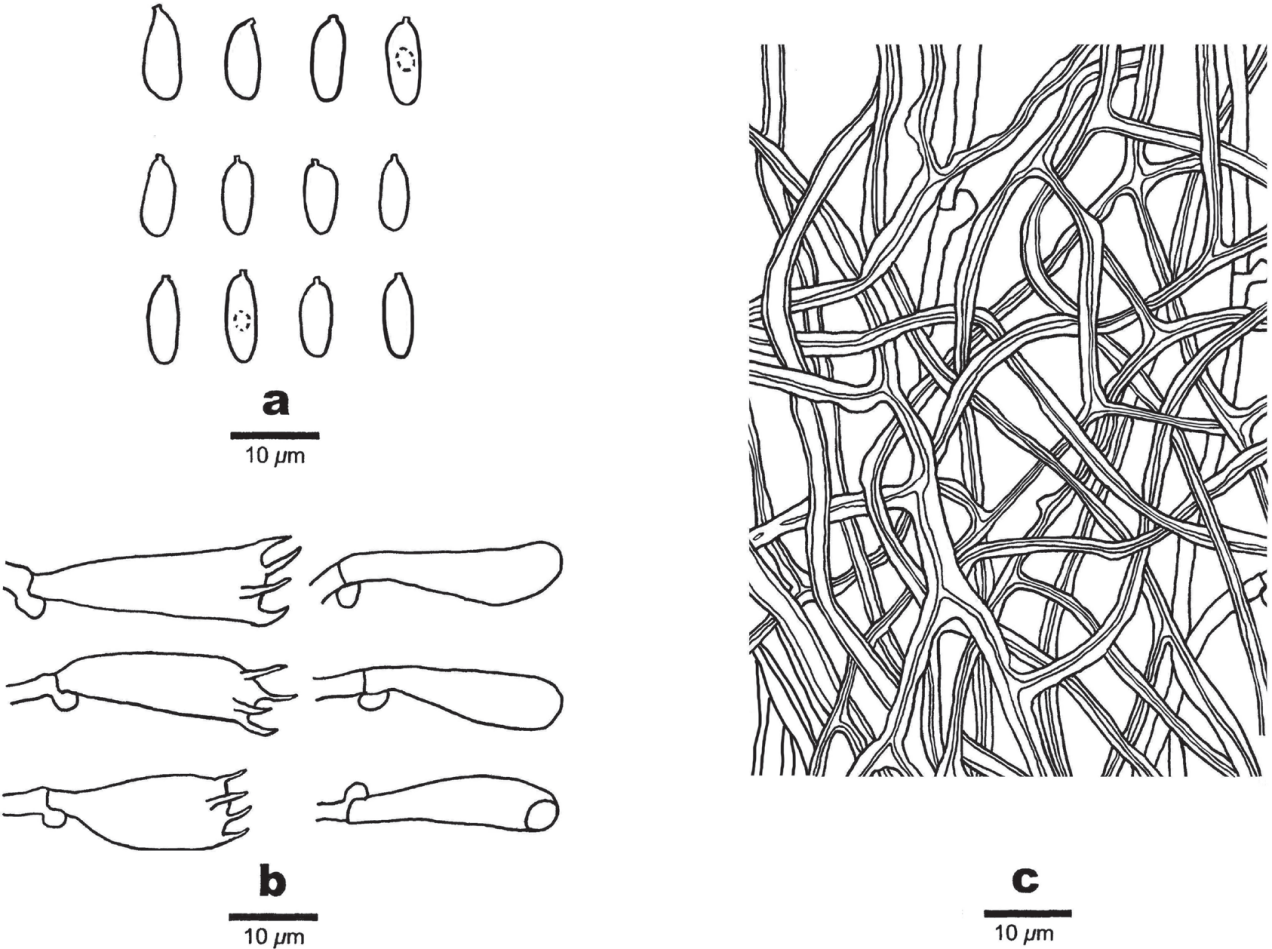
Microscopic structures of *Polyporusminutissimus* (Wu970/Wu 971) **a** basidiospores **b** basidia and basidioles **c** hyphae from trama.

***Tubes*.** Generative hyphae frequent, colorless, thin-walled, 2–3 μm in diam; skeleto-binding hyphae dominant, colorless, thick-walled with a wide lumen, moderately branched, interwoven, 1–3 μm in diam. Cystidia and cystidioles absent. Basidia clavate, with four sterigmata and a basal clamp connection, 22–28 × 7–9 μm; basidioles in shape similar to basidia, but slightly smaller.

***Stipe*.** Generative hyphae frequent, colorless, thin-walled, rarely branched, 3–4 μm in diam; skeleto-binding hyphae dominant, colorless, thick-walled with a wide lumen, moderately branched, interwoven, 1.5–4 μm in diam.

***Basidiospores*.** Basidiospores cylindrical to oblong, colorless, thin-walled, smooth, IKI–, CB–, 5–9.2(–10) × (2–)2.2–4(–4.2) μm, L = 7.30 μm, W = 3.23 μm, Q = 2.25–2.27 (n = 60/2).

##### Additional specimen examined

**(paratype).** China • Zhejiang Province, Hangzhou, Yuhang District, Luniao Town; 30°25'50"N, 119°42'38"E; 155.11 m a.s.l; on ground of bamboo forest, 9 Jun. 2023; F. Wu leg., Wu 970 (BJFC040962).

## ﻿Discussion

In this study, two new species of the Polyporales - *Cerrenacaulinicystidiata* and *Polyporusminutissimus* - are proposed based on morphological and phylogenetic evidence. Our three newly sequenced *Cerrena* samples together with three samples which were defined as *Cerrena* sp. 1 by Miettinen et al. (2023) formed an independent well-supported lineage in our phylogeny (Fig. 1). The lineage is proposed as the new species *C.caulinicystidiata*. Another lineage which is defined as *Cerrena* sp. 2 in our phylogeny is closely related to *C.caulinicystidiata*, but we didn’t collect one specimen within the lineage, so the lineage is considered to be *Cerrena* sp.

*Cerrenacaulinicystidiata* is characterized by its resupinate, sometimes effused-reflexed basidiocarps, greyish orange to brownish orange pore surface, 3–8 per mm pores, and subglobose basidiospores, 3.2–4.5 × 2.8–3.5 µm in size. *C.albocinnamomea* (Y.C. Dai & Niemelä) H.S. Yuan originally described from Northeast China resembles *C.caulinicystidiata* by sharing resupinate and easily separable basidiocarps. However, *C.albocinnamomea* differs from *C.caulinicystidiata* by its clavate to pyriform cystidia, slightly smaller ellipsoid basidiospores (2.8–3.5 × 2–3 µm vs. 3.2–4.5 × 2.8–3.5 µm), and a dimitic hyphal system (Yuan 2014).

In addition, *Rigidoporusvinctus* (Berk.) Ryvarden [≡ *Physisporinusvinctus* (Berk.) Murrill, Wu et al. 2017] resembles *C.caulinicystidiata* by having resupinate basidiocarps, ochraceous pore surface, ventricose cystidia with a subcylindric appendage, encrusted cystidia, and subglobose basidiospores, but it can be distinguished from the latter species by its smaller pores (6–12 per mm vs. 3–8 per mm) and generative hyphae with simple septa (Ryvarden and Johansen 1980).

The genus *Cerrena* is widely distributed and has diverse morphological characteristics. Currently, there are 13 records according to Index Fungorum (http://www.indexfungorum.org). However, *C.* ‘*gilbertsonii*’ Ryvarden cannot be distinguished from *C.cystidiata* Rajchenb. & De Meijer by morphological characteristics, and *C.* ‘*multipileata*’ (C.L. Leite & J.E. Wright) Miettinen cannot be distinguished from *C.zonata* (Berk.) H.S. Yuan (Miettinen et al. 2023). *Cerrenaaurantiopora* J.S. Lee & Y.W. Lim is a synonym of *C.albocinnamomea* (Lee and Lim 2010; Miettinen et al. 2023). Therefore, we provide a Key to 11 undisputed *Cerrena* species including the new species.

### ﻿Key to species of the *Cerrena*

**Table:** 

1	Paleotropical or temperate-boreal species	**2**
–	Neotropical (South American) species	**9**
2	Basidiocarp poroid, occasionally lacerate	**3**
–	Basidiocarp irpicoid	**8**
3	Pore surface umbrinous to bay or blackish	***C.subglabrescens***
–	Pore surface white, light orange to brown	**4**
4	Pores umber, round, 1–2 per mm	***C.drummondii***
–	Pores round to angular, > 3 per mm	**5**
5	Basidiospores narrowly ellipsoid, 7.5–10 × 2.5–3.5 µm	***C.caperata***
–	Basidiospores ellipsoid to broadly-ellipsoid, < 6 µm in length	**6**
6	Pores angular, dissepiments even or lacerate	***C.albocinnamomea***
–	Pores rounded to irregular	**7**
7	Basidiocarps coriaceous, imbricate	***C.fulvocinerea***
–	Basidiocarps resupinate, sometimes effused-reflexed	***C.caulinicystidiata***
8	Pore surface white to cream	***C.unicolor***
–	Pore surface first white to pale ochraceous	***C.zonata***
9	Cystidia present	***C.cystidiata***
–	Cystidia absent	**10**
10	Pore surface pale cinnamon to brown	***C.sclerodepsis***
–	Pore surface dark brown to almost black	***C.hydnoides***

In the phylogenetic analysis of *Polyporus*, *P.minutissimus* was assigned to the squamosus clade with strong support (100/1.00, Fig. 2). The squamosus clade has always been supported by phylogenetic analysis based on the ITS+nLSU or eight-gene datasets, but the species within this clade cannot be combined into a monophyletic genus because they manifest greatly diverse morphology (Ji et al. 2022). Phylogenetically, *P.minutissimus* is closely related to *P.hemicapnodes* and *P.parvovarius* (Fig. 2). *P.hemicapnodes* was described from Dolosbagey (Sri Lanka). For some time, it was treated as a synonymy of *P.leprieurii* (Núñez and Ryvarden 1995), which differs from *P.minutissimus* by its larger basidiocarps (up to 10 cm vs. up to 1.5 cm), cream to tan pore surface, and longer stipe (up to 5 cm vs. up to 0.5 cm, Berkeley and Broome 1873). *Polyporusparvovarius* has microscopic features similar to *P.minutissimus*. However, *P.parvovarius* differs by its smaller basidiocarps (up to 0.35 cm vs. up to 1.5 cm) and light buff to brown pileal surface (Tibpromma et al. 2017).

Macro-morphologically, *Polyporusminutissimus* has a depressed center or infundibuliform basidiocarps and black stipe, cream to buff yellow pileal surface, and 6–9 per mm pores. Microscopically, it has a dimitic hyphal system, strongly branched skeleton-binding hyphae in both trama and context, and cylindrical basidiospores. Morphologically, *P.lamelliporus* B.K. Cui, Xing Ji & J.L. Zhou is similar to *P.minutissimus* by sharing depressed center or infundibuliform basidiocarps, cream to buff yellow pileal surface, and similar-sized basidiospores, but the former differs through its larger basidiocarps (up to 5.2 cm vs. up to 1.5 cm), longer stipe (1–3.5 cm vs. 0.3–0.5 cm), and larger pores (0.5–1 per mm vs. 6–9 per mm, Ji et al. 2022). In addition, *Picipesbaishanzuensis* J.L. Zhou & B.K. Cui, which is similar to *P.minutissimus* and shares infundibuliform basidiocarps and a black stipe, has also been reported from Baishanzu nature reserve, which is the type producing area of our new species. However, *P.baishanzuensis* differs from *P.minutissimus* by its larger basidiocarps (up to 5.5 cm vs. up to 1.5 cm) and smaller basidiospores (6.6–7.9 × 2.5–3.1 µm vs. 5–9.2 × 2.2–4 µm; Zhou et al. 2016).

*Polyporus* is a very complicated genus with more than 3000 records according to the Index Fungorum. However, studies on *Polyporus* species in China are gradually being carried out, with some Chinese species having been described in Cui et al. (2019) and Ji et al. (2022). Therefore, we provide a Key to Chinese *Polyporus* species including the new species.

### ﻿Key to species of *Polyporus* in China

**Table:** 

1	Stipe absent	***P.megasporoporus***
–	Stipe present	**2**
2	Stipe bearing black cuticle	**3**
–	Stipe white to ochraceous	**7**
3	Pileal surface covered with dark-brown to reddish-brown squamules	***P.squamosus***
–	Pileal surface glabrous	**4**
4	Pores more than 5 per mm	**5**
–	Pores less than 5 per mm	**6**
5	Pileal surface concentrically zonate; basidiospores 5.4–7.6 × 2.9–3.8 μm	***P.hemicapnodes***
–	Pileal surface azonate; basidiospores 7.5–9 × 2.5–3.3 μm	***P.varius***
6	Pores 3–5 per mm	***P.mangshanensis***
–	Pores 1–2 per mm	***P.subvarius***
7	Stipes numerous and branched	***P.umbellatus***
–	Stipes usually single and not branched	**8**
8	Basidiospores < 8 μm in length	**9**
–	Basidiospores > 8 μm in length	**11**
9	Basidiocarps imbricate	***P.hapalopus***
–	Basidiocarps solitary	**10**
10	Pores angular, 2–3 per mm	***P.brumalis***
–	Pores round, 4–5 per mm	***P.ciliatus***
11	Pileal surface with radial stripes	**12**
–	Pileal surface without radial stripes	**13**
12	Pores 2–5 per mm	***P.cuticulatus***
–	Pores 6–9 per mm	***P.minutissimus***
13	Basidiospores usually < 10 μm in length	**14**
–	Basidiospores usually > 10 μm in length	***P.tuberaster***
14	Cystidioles absent	**15**
–	Cystidioles infrequent	***P.austrosinensis***
15	Basidia < 27 μm in length	**16**
–	Basidia > 27 μm in length	***P.lamelliporus***
16	Basidiospores smaller, 6–8.3 × 2.2–3 μm	***P.arcularius***
–	Basidiospores larger, 7.7–10 × 3–3.9 μm	***P.auratus***

Polyporales is a large group of Basidiomycota with diverse morphology and phylogeny. There have been over 577 taxonomic proposals in the Polyporales and 2,183 publications with the keyword ‘Polyporales’ over the past decade (Binder et al. 2013; Justo et al. 2017). However, the species in the order are still not sufficiently investigated in Asia, especially in the subtropics and tropics (Li et al. 2016; Hyde 2022). New DNA sequencing techniques have revolutionized fungal taxonomy and diversity, with multi-marker datasets. In the present study, two new polypore species, *C.caulinicystidiata* and *P.minutissimus* were found in subtropical regions, which enriches our understanding of the fungal diversity of the Polyporales in Asia.

## Supplementary Material

XML Treatment for Cerrena caulinicystidiata

XML Treatment for Polyporus minutissimus

## ﻿Additional information

### Author ORCIDs

Zi-Wei Zheng https://orcid.org/0009-0006-8442-408X

Qiu-Yue Zhang https://orcid.org/0000-0001-9458-3566

Hai-Sheng Yuan https://orcid.org/0000-0001-7056-140X

Fang Wu https://orcid.org/0000-0002-1455-6486

### Data availability

All of the data that support the findings of this study are available in the main text or Supplementary Information.

## Supplementary materials

Supplementary material 1 31102 Treebase Data type nxs https://binary.pensoft.net/file/1068250This dataset is made available under the Open Database License (http://opendatacommons.org/licenses/odbl/1.0/). The Open Database License (ODbL) is a license agreement intended to allow users to freely share, modify, and use this Dataset while maintaining this same freedom for others, provided that the original source and author(s) are credited.Zi-Wei Zheng, Qiu-Yue Zhang, Li-Rong Zhang, Hai-Sheng Yuan, Fang Wu

Supplementary material 2 31103 Treebase Data type nxs https://binary.pensoft.net/file/1068251This dataset is made available under the Open Database License (http://opendatacommons.org/licenses/odbl/1.0/). The Open Database License (ODbL) is a license agreement intended to allow users to freely share, modify, and use this Dataset while maintaining this same freedom for others, provided that the original source and author(s) are credited.Zi-Wei Zheng and Qiu-Yue Zhang, Li-Rong Zhang, Hai-Sheng Yuan, Fang Wu
